# Safety and efficacy of chocolate balloon in the treatment of infrapopliteal artery disease

**DOI:** 10.1186/s42155-024-00501-2

**Published:** 2025-01-07

**Authors:** Ridong Wu, Qingqi Yang, Mian Wang, Zilun Li, Chen Yao, Guangqi Chang

**Affiliations:** Department of Vascular Surgery, The First Affiliated Hospital, Sun Yat-Sen University, Guangzhou, China

## Abstract

**Objective:**

To evaluate the safety and efficacy of chocolate balloons in patients with chronic limb-threatening ischemia (CLTI) and infrapopliteal artery disease, and compare them with conventional balloons.

**Methods:**

This single-center retrospective study included 167 patients with CLTI and infrapopliteal who underwent endovascular intervention with or without chocolate balloons from September 1, 2019 to June 30, 2023. The primary endpoint was amputation-free survival (AFS). Secondary endpoints included major amputation, the absence of clinically driven target lesion revascularization (CD-TLR), the incidence of flow-limiting dissection, below-the-knee (BTK) stent implantation, change in Rutherford clinical grade, procedural success, and major adverse cardiovascular events (MACEs). Patients were followed at 30 days, 6 months, and 12 months to assess symptom improvement, vascular patency as determined by dual-function ultrasound or angiography, and survival.

**Results:**

At 12 months, AFS was noted in 78.1% of patients in chocolate balloon group and 70.7% of those in conventional balloon group (*p* = 0.37). The chocolate balloon group demonstrated a significantly higher rate of CD-TLR absence, with 84.0% compared to 69.4% in the conventional balloon group (*p* = 0.04). The chocolate balloon group had a major amputation-free rate of 92.8%, slightly better than the 89.5% in the conventional balloon group (*p* = 0.58).

Notably, the chocolate balloon group significantly reduced flow-limiting dissection (*p* = 0.02) and BTK stent implantation (*p* = 0.03) compared to the conventional balloon group.

**Conclusion:**

Chocolate balloon reduces the incidence of flow-limiting dissection and BTK stent implantation in patients with CLTI and infrapopliteal. Compared with conventional balloons, there was less lesion revascularization at 12 months, but no significant benefit was found in improving ASF and reducing major amputation of the affected limb.

**Supplementary Information:**

The online version contains supplementary material available at 10.1186/s42155-024-00501-2.

## Introduction

The lower limb peripheral arterial disease (PAD) is characterized by atherosclerotic occlusive lesions in the arteries of the lower extremities. As a significant global public health concern affecting more than 200 million individuals [1], it not only results in impaired limb function but also diminishes overall quality of life [2, 3]. Moreover, it is believed to be associated with an elevated risk of major adverse cardiovascular events (MACEs)[4]. The critical limb-threatening ischemia (CLTI), as the advanced stage of PAD, poses a significant risk, particularly in terms of major amputation and mortality [5, 6]. The development of CLTI is undoubtedly influenced by multiple factors, however, the rise in infrapopliteal artery disease contributes to the occurrence of amputation and remains a significant obstacle for effective endovascular treatment [7].

The use of plain old balloon angioplasty (POBA) has been extensively employed in the treatment of PAD, including infrapopliteal artery disease, due to its broad applicability and practicality [8]. A meta-analysis conducted by Romiti et al. reported an immediate technical success rate as high as 89.0 ± 2.2% for infrapopliteal artery disease, while the average patency rate of conventional balloon at 1 year was found to be 58.1 ± 4.6%, accompanied by a limb amputation of 14.0% [9]. Long-term patency following conventional balloon remain unsatisfactory, particularly in cases involving long-segment lesions and chronic total occlusions (CTO)[10]. Furthermore, elastic recoil and flow-limited dissection are common occurrences during procedures that can restrict its therapeutic efficacy [11].

The Chocolate balloon, a Nitinol stent constrained standard balloon, is designed to expand blood vessels evenly while minimizing damage during balloon dilation [12, 13]. Recent studies have shown that chocolate balloon have exhibited exceptional surgical outcomes, a high rate of long-term patency, and a low occurrence of dissection in the superficial femoral artery (SFA) and popliteal artery (PA) [14, 15]. The findings of another study also demonstrated the superiority of chocolate balloon over conventional balloon in vascular preparation for SFA and PA lesions treated with drug-coated balloon (DCB) [16]. However, there are few reports on the use of chocolate balloons for infrapopliteal artery disease. Hence, this study aims to compare the efficacy of chocolate balloons with conventional balloon for infrapopliteal artery disease in a population of patients with CLTI, aiming to evaluate the safety and effectiveness in real-world scenarios.

## Materials and Methods

### Patients

Between September 1, 2019, and June 30, 2023, consecutive patients with infrapopliteal artery disease and CLTI (Rutherford class 4–6) who underwent endovascular interventions were included in this single-center, retrospective cohort study. All included patients were assigned to the same treatment group, with the procedures performed by a chief physician with 20 years of experience in vascular surgery, employing the same treatment strategy for all lesions. The use of chocolate balloons is entirely determined by the operator based on clinical judgment and the anatomical characteristics of the lesions, such as length, type, and vessel diameter.

Exclusion criteria were as follows: life expectancy < 1 year, presence of acute thrombosis in the target vessel, planned major amputation of the target limb, patients who had received other treatments for the target lesions, including drug-coated balloons or bypass surgery. A total of 167 patients were enrolled. Demographic information including medical history, physical examination, ankle-brachial index (ABI) and imaging examination were collected upon admission. Rutherford and Fontaine classifications were assigned based on patient conditions to assess severity. WIfI classification was established according to the Vascular Surgery Society classification system for limbs at risk, which included wound extent, ischemia degree and infection extent [17]. The information pertaining to the target lesion, including lesion location, length, type (de novo or restenosis), lesion characteristics (stenosis or CTO), degree of calcification, flow-limiting dissection, and intervention in inflow and outflow tracts was retrospectively obtained through angiography analysis, surgical records review, and imaging examinations. The study was conducted in accordance with the principles of the Helsinki Declaration and was approved by the ethics committee. Due to the study's retrospective nature, the need for patient consent was waived.

## Interventions

Access was gained via antegrade femoral artery puncture, and a 6F arterial sheath (Terumo, Tokyo, Japan) was inserted. Heparin (60 U/kg) was administered intravenously. Angiography through the arterial sheath showed infrapopliteal artery disease. As many infrapopliteal arteries as possible were opened. Using a guidewire (V-18 control guidewire; Boston Scientific, Boston Scientific, Massachusetts, USA) and catheter (Cordis, Santa Clara, CA, USA), the target infrapopliteal artery was selectively engaged under roadmap guidance. The guidewire and catheter were used to cross the lesion, and the true distal lumen was reached. A 2.5 × 120 mm or 3.0 × 120 mm chocolate balloon (TriReme Medical LLC, Pleasanton, CA-USA) was selected based on the target artery diameter. The balloon was advanced across the lesion, and using a pressure injector, the pressure was slowly increased to 4 atm over 30 s to expand and shape the constrained balloon. The pressure was then slowly increased to 12 atm and maintained for 3 min before deflation. Vessel expansion was performed from distal to proximal. If the desired expansion was not achieved, the original balloon was re-used and dilated for up to 5 min until the optimal result was obtained. If persistent residual stenosis (> 50%) or severe flow-limiting dissection remained, BTK stent implantation was performed. The same treatment protocol was applied to the conventional balloon group. The representative cases can be found in the supplementary materials.

## Study endpoints and follow up

The primary endpoint was amputation-free survival (AFS) (defined as freedom from above-ankle amputation or death from any cause). The secondary endpoints included freedom from CD-TLR and major amputation (above the ankle), the incidence of flow-limiting dissection, BTK stent implantation, changes in Rutherford clinical classification, and procedural success defined as residual diameter stenosis ≤ 30% without the occurrence of a severe flow-limiting dissection and MACEs. MACEs were defined as a composite of cardiovascular mortality, myocardial infarction (MI), stroke or transient ischemic attack (TIA), and heart failure (HF) or hospitalization for heart failure (HHF). Patients were followed up at 30 days, 6 months, and 12 months postoperatively to assess symptom improvement, vessel patency determined by duplex ultrasonography or angiography, and survival.

## Statistical analysis

The continuous variables were presented as mean ± standard deviation or median (interquartile range). Comparisons of continuous variables were conducted using T-tests or Mann–Whitney U tests. Categorical variables were expressed as counts (%), and chi-square tests were employed for comparisons. Kaplan–Meier methods were used to estimate the rates of survival, AFS and major amputation during the 12-month follow-up period. Censoring was defined as death or dropout, and differences between groups were assessed using the log-rank test. All analysis with P < 0.05 for statistical significance. Statistical analysis was performed with IBM SPSS 25.0 software (IBM Corp., Armonk, NY, USA) and GraphPad Prism 10.0 (GraphPad Software, Inc., San Diego, CA).

## Result

### Patient and Lesion Characteristics

**Fig. 1:**
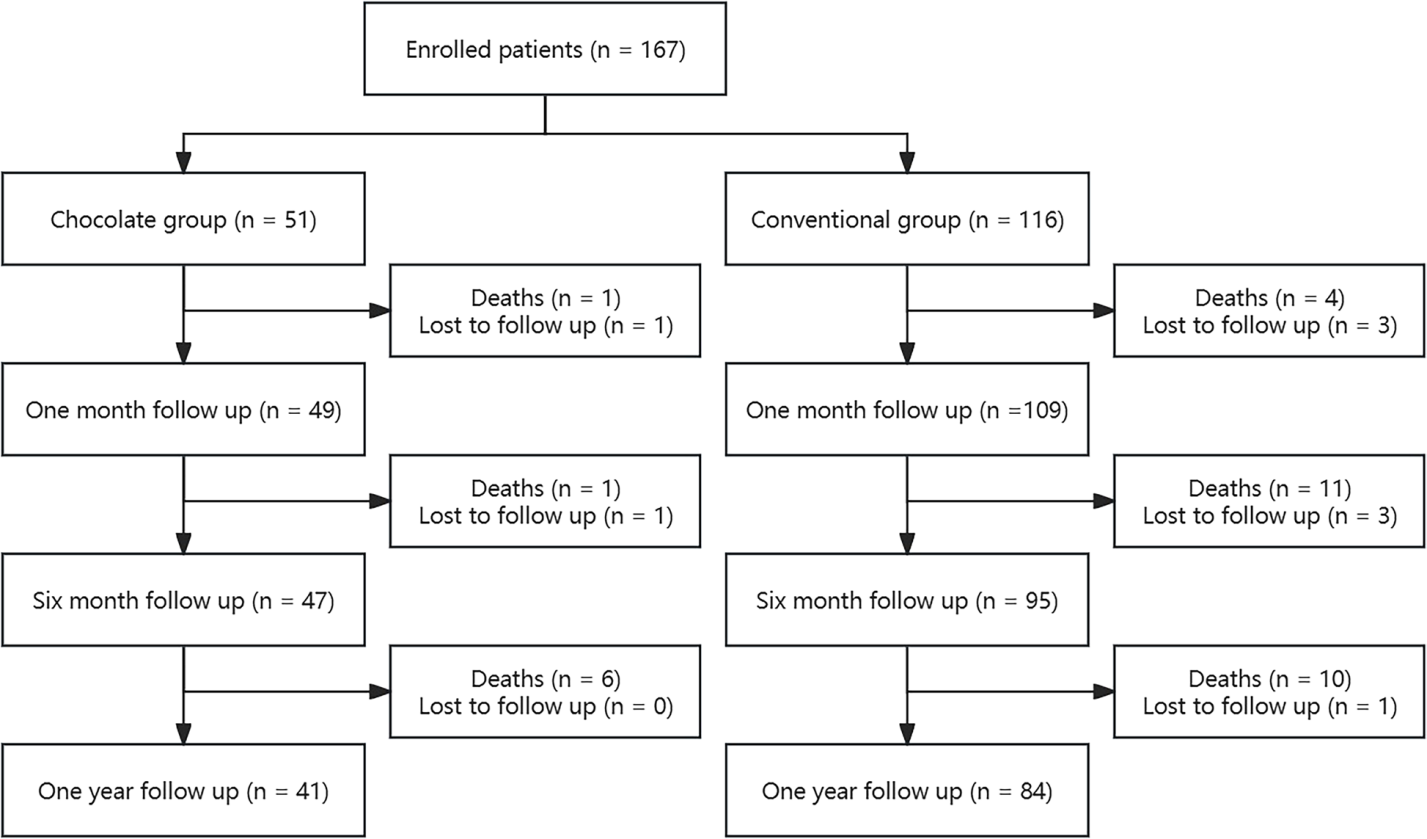
Flow diagram of patients with infrapopliteal artery disease and chronic limb-threatening ischemia undergoing endovascular intervention with chocolate balloon or conventional balloon and their one-year follow-up

Patient enrollment and 12-month follow-up as shown in Fig. 1, a total of 167 patients with CLTI and infrapopliteal artery disease were enrolled in this study. 51 patients received chocolate balloon and 116 patients received conventional balloon. As shown in Table 1, there was no significant difference in baseline characteristics between the two groups of patients. As shown in Table 2, a total of 177 limbs were treated with intervention. There is no significant difference in lesions and procedural characteristics between the two groups.

**Table 1:** Baseline characteristics of 167 patients with peripheral artery disease treated with chocolate or conventional balloons

	**Chocolate group (*n* = 51 patients; 57 limbs)**	**Conventional group (*n* = 116 patients; 120 limbs)**	***p* value**
**Clinical characteristics**			
Age—years	72.2 $\pm$ 9.9	70.9 $\pm$ 8.4	0.40
Height—cm	165.0 $\pm$ 6.8	163.8 $\pm$ 7.9	0.34
Weight—kg	61.9 $\pm$ 8.9	59.4 $\pm$ 10.3	0.13
Male (%)	38(74.5)	83(71.6)	0.69
Somking (%)	29(56.9)	50(43.1)	0.11
Diabetes (%)	31(60.8)	54(48.3)	0.09
Hypertension (%)	39(76.5)	78(46.6)	0.23
Coronary artery disease (%)	10(19.6)	25(21.6)	0.78
Hyperlipidaemia (%)	11(21.6)	29(25.0)	0.63
Taget limb characteristics			
Rutherford class			0.62
4 (%)	17(29.8)	43(35.8)	
5 (%)	31(54.4)	63(52.5)	
6 (%)	9(15.8)	14(11.7)	
Fontaine class			0.67
3 (%)	19(33.3)	44(36.7)	
4 (%)	38(66.7)	76(63.3)	
WIfI stage in patients with CLTI			0.26
1 (%)	6(10.5)	11(9.1)	
2 (%)	21(36.8)	29(24.2)	
3 (%)	20(35.1)	47(39.2)	
4 (%)	10(17.5)	33(27.5)

**Table 2:** Lesions and procedural characteristics of 167 patients with peripheral artery disease treated with chocolate or conventional balloons

	**Chocolate group (*n* = 51 patients; 57 limbs)**	**Conventional group (*n* = 116 patients; 120 limbs)**	***p*** value
**Target lesion characteristics**			
Lesion type (%)			0.82
De novo lesion	51(89.4)	106(88.3)	
Re-stenotic lesion	6(10.5)	14(11.7)	
Chronic occlusions (%)	35(61.4)	82(68.3)	0.36
Calcification			0.16
None (%)	10(17.5)	24(20.0)	
Mild (%)	19(33.3)	42(35.0)	
Moderate (%)	21(36.8)	27(22.5)	
Severe (%)	7(12.3)	27(22.5)	
Lesion location			
Anterior tibial (%)	55(96.4)	116(96.7)	0.95
Tibioperoneal trunk (%)	25(43.9)	49(40.8)	0.70
Posterior tibial (%)	49(86.0)	100(83.3)	0.65
Peroneal (%)	37(64.9)	86(71.7)	0.36
Lesion length (IQR)—cm	137(64.0)	127(88.0)	0.38
**Procedural characteristics**			
Treated vessels -n			0.72
1 vessel intervention (%)	25(43.9)	52(43.3)	
2 vessel intervention (%)	25(43.9)	55(45.8)	
3 vessel intervention (%)	7(12.2)	11(9.2)	
4 vessel intervention (%)	0(0.0)	2(1.7)	
Maximum balloon diameter (IQR)—mm	3(0.5)	3(0.5)	0.99
Concomitant inflow intervention (%)	18(31.6)	56(46.7)	0.06
Concomitant outflow intervention, below the ankle (%)	12(21.1)	26(21.7)	0.93
Intraprocedural use of thrombolysis (%)	3(5.3)	12(10.0)	0.29
Ankle-brachial index			
Pre-intervention (IQR)	0.58(0.50)	0.51(0.74)	0.35
Post-intervention (IQR)	0.96(0.19)	0.98(0.30)	0.68
MACEs (%)	3(5.9)	9(7.8)	0.67

## Acute outcomes for all treated vessels

For the chocolate balloon group, compared to the conventional balloon group, they have significantly fewer flow-limiting dissections (1.0% vs 7.9%, p = 0.02), and also require fewer implantation of BTK stents (2.1% vs 8.9%, p = 0.03). There is no significant difference in procedural success between the two groups. (Table 3).

**Table 3:** Acute outcomes of all vessels treated with chocolate or conventional balloons

	**Chocolate group (n = 96 Vessels)**	**Conventional group (n = 203 Vessels)**	***p* value**
BTK stent implantation (%)	2(2.1)	18(8.9)	0.03
Flow-limiting dissection (%)	1(1.0)	16(7.9)	0.02
Procedural success (%)	91(94.8)	183(90.1)	0.18

## Efficacy and safety results at 12 months of follow-up

**Fig. 2:**
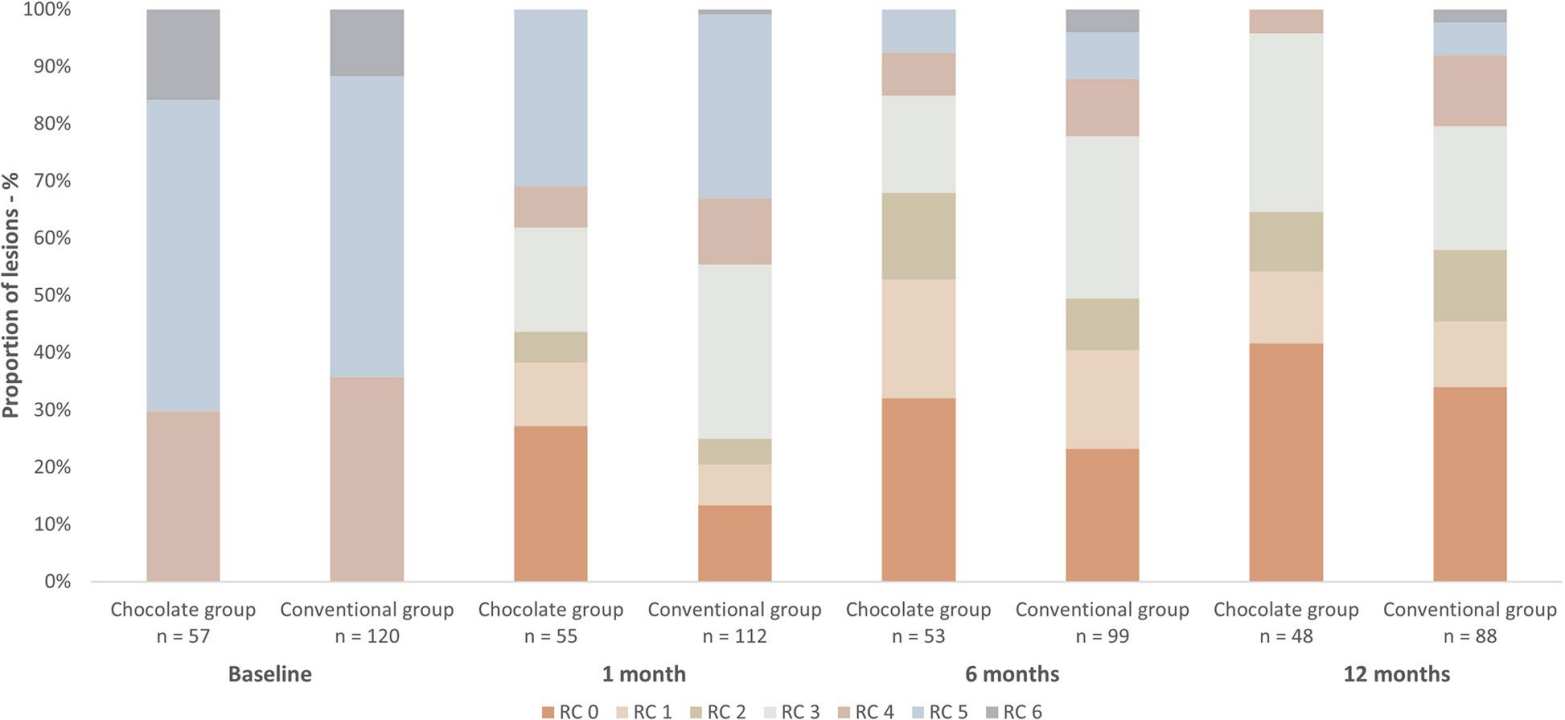
Distribution of Rutherford clinical categories at baseline, 1, 6 and 12 months

Figure 2 shows the distribution of Rutherford clinical classification for patients with CLTI and infrapopliteal artery disease who underwent endovascular intervention using a chocolate balloon or a conventional balloon at baseline, 1 month, 6 months, and 12 months.

**Fig. 3:**
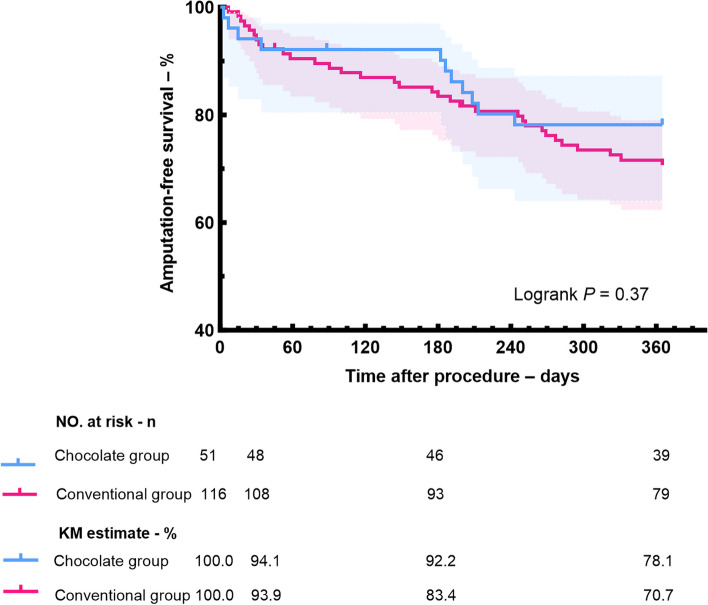
Kaplan–Meier estimates of amputation-free survival in chocolate balloon group and conventional balloon group

**Fig. 4:**
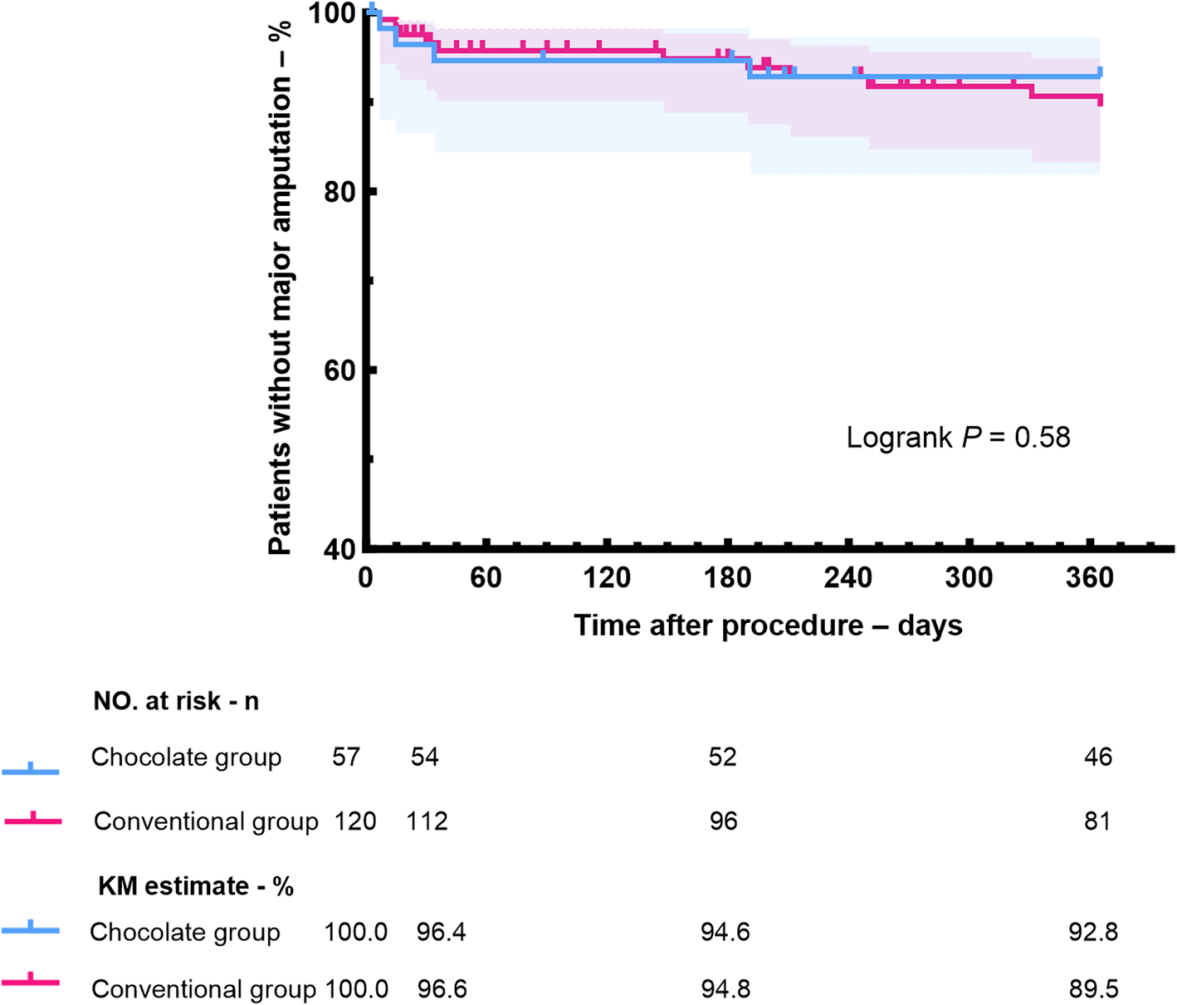
Kaplan–Meier estimates of freedom from major amputation in chocolate balloon group and conventional balloon group

**Fig. 5:**
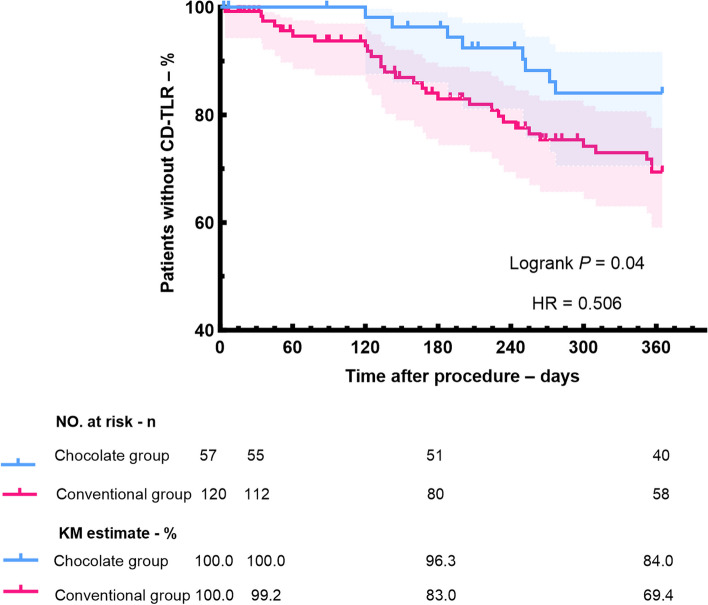
Kaplan–Meier estimates of freedom from CD-TLR in chocolate balloon group and conventional balloon group

Although there were no significant differences between the two groups in the 12-month rates of AFS (Fig. 3), major amputation (Fig. 4), the chocolate balloon group exhibited slightly better performance compared to the conventional balloon group (AFS: 78.1% vs 70.7%, p = 0.37; Freedom from major amputation: 92.8% vs 89.5%, p = 0.58). Notably, the rates of freedom from CD-TLR at 12 months was 84.0% in the chocolate balloon group and 69.4% in the conventional group, with a statistically significant difference between the two groups (log-rank p = 0.04) (Fig. 5).

## Discussion

This study is the first to evaluate the safety and efficacy of chocolate balloon in infrapopliteal artery disease, while comparing it with conventional balloon. As reported in the studies on the use of chocolate balloon for ATK lesions, it has been proven to reduce the occurrence of flow-limiting dissections and the need for stent implantation [12–16]. Echoing these findings, this study corroborates the benefits of the chocolate balloon in a different yet equally critical vascular territory. Compared to the conventional balloon group at 90.1%, the chocolate balloon group achieved a 94.8% procedural success (immediate residual stenosis ≤ 30% and no flow-limiting dissection), with a significantly lower incidence of flow-limiting dissections and BTK stent implantation, even in cases involving longer lesions, typically associated with more complex and challenging scenarios.

This study also observed that at 1 month, 6 months, and 12 months postoperatively, the AFS in the chocolate balloon group were 94.1%, 92.2%, and 78.1%, respectively, all of which were superior to those of the conventional balloon group during the same periods. Although not statistically significant, the trend suggests that the chocolate balloon may have clinical efficacy in the short to medium term.

As the most serious stage of peripheral artery disease, CLTI has a poor long-term prognosis. It has been reported that the 1-year amputation of CLTI patients usually exceeds 15% to 20%, and the 1-year mortality is in the range of 15% to 40%. Moreover, the risks continue to increase with the increase in disease severity and the presence of other comorbidities (such as diabetes) [18–20]. This study also found these trends, with a 1-year major amputation was 18.5%. Besides, we found that chocolate balloons coule effectively reduce the reconstruction, and 84.0% of the affected limbs were free of CD-TLR at 12 months, which was significantly better than conventional balloons We speculate that this may be related to the higher technical success rate of chocolate balloons in dilating target vessels, lower incidences of flow-limiting dissection and BTK stent implantation.

It is important to note that the chocolate balloon is not yet routinely used for the treatment of infrapopliteal artery disease. Although the clinical observations in this study found that it had better efficacy than the conventional balloon and resulted in fewer cases of flow-limiting dissection and provisional stent implantation, in the long term, its limb salvage rate and survival rate were not significantly superior to those of the conventional balloon. The safety differences between the chocolate balloon and the conventional balloon remain to be discussed.

## Limitations

This study has limitations. Given its retrospective nature, small sample size, and single-center design, the generalizability of the findings to other centers may be limited. Besides, the follow-up duration in this study was restricted to 1 year only; therefore, further long-term follow-up is warranted to validate the efficacy.

## Conclusion

The use of chocolate balloons had been shown to decrease the occurrence of flow-limiting dissection and BTK stent implantation in patients with CLTI and infrapopliteal. In comparison to conventional balloons, there was a lower rate of lesion revascularization, although no significant advantage was observed in developing ASF or reducing major amputation.

## Supplementary Information

Supplementary Material 1.

## References

[1] Fowkes FG, Rudan D, Rudan I, et al. Comparison of global estimates of prevalence and risk factors for peripheral artery disease in 2000 and 2010: a systematic review and analysis. Lancet. 2013;382(9901):1329–40. 10.1016/S0140-6736(13)61249-0. 23915883 10.1016/S0140-6736(13)61249-0

[2] McDermott MM, Guralnik JM, Tian L, et al. Baseline functional performance predicts the rate of mobility loss in persons with peripheral arterial disease. J Am Coll Cardiol. 2007;50(10):974–82. 10.1016/j.jacc.2007.05.030. 17765125 10.1016/j.jacc.2007.05.030 PMC2645658

[3] McDermott MM, Liu K, Greenland P, et al. Functional decline in peripheral arterial disease: associations with the ankle brachial index and leg symptoms. JAMA. 2004;292(4):453–61. 10.1001/jama.292.4.453. 15280343 10.1001/jama.292.4.453

[4] Cook IO, Chung J. Contemporary Medical Management of Peripheral Arterial Disease. Cardiovasc Drugs Ther; 2023. Published online November 2. 10.1007/s10557-023-07516-2 . 10.1007/s10557-023-07516-2 37914901

[5] Berchiolli R, Bertagna G, Adami D, Canovaro F, Torri L, Troisi N. Chronic Limb-Threatening Ischemia and the Need for Revascularization. J Clin Med. 2023;12(7):2682. Published 2023 Apr 4. 10.3390/jcm12072682. 10.3390/jcm12072682 PMC10095037 37048765

[6] Conte MS, Bradbury AW, Kolh P, et al. Global vascular guidelines on the management of chronic limb-threatening ischemia [published correction appears in J Vasc Surg. 2019;70(2):662. 10.1016/j.jvs.2019.06.102. J Vasc Surg. 2019;69(6S):3S-125S.e40. 10.1016/j.jvs.2019.02.016. 10.1016/j.jvs.2019.02.016 PMC8365864 31159978

[7] Graziani L, Jaff MR. Drug-eluting balloons: are these failed solutions for the treatment of below-the-knee peripheral artery disease? Ann Vasc Surg. 2014;28(4):1078–9. 10.1016/j.avsg.2014.02.002. 24530717 10.1016/j.avsg.2014.02.002

[8] Söder HK, Manninen HI, Jaakkola P, et al. Prospective trial of infrapopliteal artery balloon angioplasty for critical limb ischemia: angiographic and clinical results. J Vasc Interv Radiol. 2000;11(8):1021–31. 10.1016/s1051-0443(07)61332-3. 10997465 10.1016/s1051-0443(07)61332-3

[9] Romiti M, Albers M, Brochado-Neto FC, Durazzo AE, Pereira CA, De Luccia N. Meta-analysis of infrapopliteal angioplasty for chronic critical limb ischemia. J Vasc Surg. 2008;47(5):975–81. 10.1016/j.jvs.2008.01.005. 18372148 10.1016/j.jvs.2008.01.005

[10] El Khoury R, Brodmann M, Schneider PA. Progress on developing an effective below-the-knee drug-coated balloon. Rev Cardiovasc Med. 2021;22(3):585–95. 10.31083/j.rcm2203070. 34565062 10.31083/j.rcm2203070

[11] Karnabatidis D, Spiliopoulos S, Katsanos K, Siablis D. Below-the-knee drug-eluting stents and drug-coated balloons. Expert Rev Med Devices. 2012;9(1):85–94. 10.1586/erd.11.67. 22145843 10.1586/erd.11.67

[12] Spiliopoulos S, Karamitros A, Reppas L, Brountzos E. Novel balloon technologies to minimize dissection of peripheral angioplasty. Expert Rev Med Devices. 2019;16(7):581–8. 10.1080/17434440.2019.1626715. 31149847 10.1080/17434440.2019.1626715

[13] Schillinger M, Minar E. Percutaneous treatment of peripheral artery disease: novel techniques. Circulation. 2012;126(20):2433–40. 10.1161/CIRCULATIONAHA.111.036574. 23147770 10.1161/CIRCULATIONAHA.111.036574

[14] Mustapha JA, Lansky A, Shishehbor M, et al. A prospective, multi-center study of the chocolate balloon in femoropopliteal peripheral artery disease: The Chocolate BAR registry. Catheter Cardiovasc Interv. 2018;91(6):1144–8. 10.1002/ccd.27565. 29513389 10.1002/ccd.27565

[15] Sirignano P, Mansour W, d’Adamo A, Cuozzo S, Capoccia L, Speziale F. Early Experience with a New Concept of Angioplasty Nitinol-Constrained Balloon Catheter (Chocolate®) in Severely Claudicant Patients. Cardiovasc Intervent Radiol. 2018;41(3):377–84. 10.1007/s00270-017-1840-9. 29159684 10.1007/s00270-017-1840-9

[16] Shirai S, Mori S, Yamaguchi K, et al. Impact of Chocolate percutaneous transluminal angioplasty balloon on vessel preparation in drug-coated balloon angioplasty for femoropopliteal lesion. CVIR Endovasc. 2022;5(1):46. Published 2022 Sep 1. 10.1186/s42155-022-00324-z. 10.1186/s42155-022-00324-z PMC9437152 36048380

[17] Mills JL Sr, Conte MS, Armstrong DG, et al. The Society for Vascular Surgery Lower Extremity Threatened Limb Classification System: risk stratification based on wound, ischemia, and foot infection (WIfI). J Vasc Surg. 2014;59(1):220–34.e342. 10.1016/j.jvs.2013.08.003. 24126108 10.1016/j.jvs.2013.08.003

[18] Duff S, Mafilios MS, Bhounsule P, Hasegawa JT. The burden of critical limb ischemia: a review of recent literature. Vasc Health Risk Manag. 2019;15:187-208. Published 2019 Jul 1. 10.2147/VHRM.S209241. 10.2147/VHRM.S209241 PMC6617560 31308682

[19] Melillo E, Micheletti L, Nuti M, et al. Long-term clinical outcomes in critical limb ischemia–A retrospective study of 181 patients. Eur Rev Med Pharmacol Sci. 2016;20(3):502–8. 26914126

[20] Vogel TR, Dombrovskiy VY, Carson JL, Graham AM. In-hospital and 30-day outcomes after tibioperoneal interventions in the US Medicare population with critical limb ischemia. J Vasc Surg. 2011;54(1):109–15. 10.1016/j.jvs.2010.12.055. 21397441 10.1016/j.jvs.2010.12.055

