# Supplementary material for: Safety and efficacy of chocolate balloon in the treatment of infrapopliteal artery disease
Source: CVIR Endovasc. 2025 Jan 7;8:3. doi: 10.1186/s42155-024-00501-2 (PMC11707162; doi:10.1186/s42155-024-00501-2)
Supplement: Supplementary file 1 — Supplementary Material 1. [file 42155_2024_501_MOESM1_ESM.docx]

Supplementary material


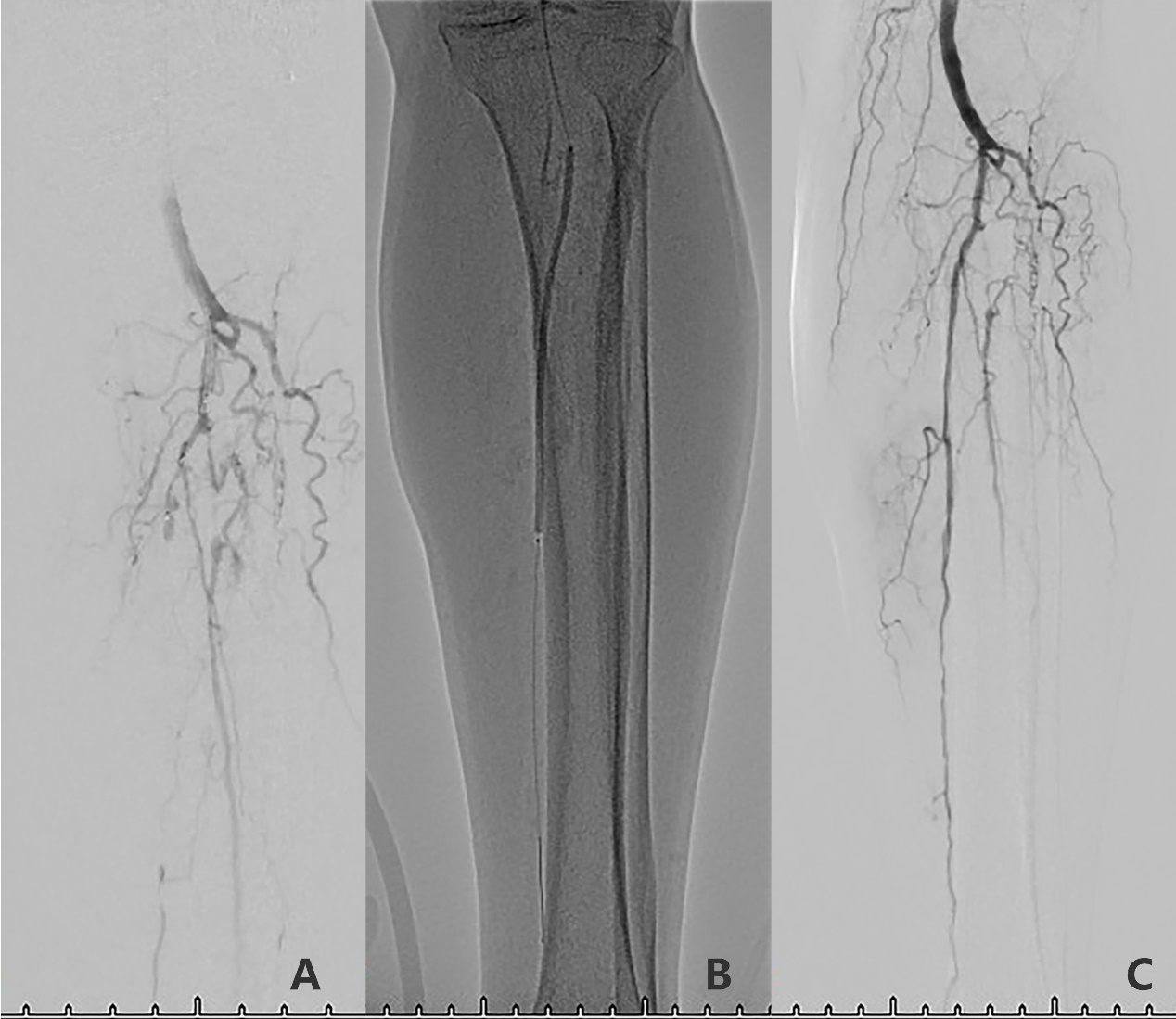


Figure S1. Representative case. Intraoperative angiography indicated severe stenosis in the proximal segment of the posterior tibial artery and occlusion in the mid-segment (A). After the guidewire passed through the lesion, a 2.5 mm chocolate balloon was used to dilate the lesion (B). Post-dilation angiography showed smooth blood flow in the mid and upper segments of the posterior tibial artery, with no dissection observed (C).
